# Prognostic Value of Apoptosis-Inducing Factor (AIF) in Germ Cell Tumors

**DOI:** 10.3390/cancers13040776

**Published:** 2021-02-13

**Authors:** Katarina Letkovska, Pavel Babal, Zuzana Cierna, Silvia Schmidtova, Veronika Liskova, Katarína Kalavska, Vera Miskovska, Samuel Horak, Katarina Rejlekova, Michal Chovanec, Jozef Mardiak, Pavel Janega, Michal Mego

**Affiliations:** 1 Department of Pathology, Faculty of Medicine, Comenius University, 813 72 Bratislava, Slovakia; k.letkovska@fmed.uniba.sk (K.L.); pavel.babal@fmed.uniba.sk (P.B.); ciernaz@fmed.uniba.sk (Z.C.); samuel.horak@fmed.uniba.sk (S.H.); pavol.janega@fmed.uniba.sk (P.J.); 2 Department of Pathology, Faculty Hospital, A. Zarnova, 917 75 Trnava, Slovakia; 3 Translational Research Unit, 2nd Department of Oncology, Comenius University, Faculty of Medicine, National Cancer Institute, 833 10 Bratislava, Slovakia; silvia.schmidtova@savba.sk (S.S.); katarina.hainova@savba.sk (K.K.); 4 Department of Molecular Oncology, Cancer Research Institute, Biomedical Research Center of the Slovak Academy of Sciences, 814 39 Bratislava, Slovakia; 5 Institute of Clinical and Translational Research, Biomedical Research Center of the Slovak Academy of Sciences, 845 05 Bratislava, Slovakia; veronika.liskova@savba.sk; 6 1st Department of Oncology, Comenius University, Faculty of Medicine, St. Elisabeth Cancer Institute, 812 50 Bratislava, Slovakia; vmiskovs@ousa.sk; 7 2nd Department of Oncology, Comenius University, Faculty of Medicine, National Cancer Institute, 833 10 Bratislava, Slovakia; katarina.rejlekova@nou.sk (K.R.); michal.chovanec@nou.sk (M.C.); jozef.mardiak@nou.sk (J.M.)

**Keywords:** testicular germ cell tumors, apoptosis, apoptosis inducing factor, prognosis

## Abstract

**Simple Summary:**

Germ cell tumors (GCTs) are the most common solid malignancies in young men. GCTs are extraordinary sensitive to chemotherapy and represent a model of curable cancer. However, in a small proportion of patients the disease progresses or relapses despite administration of salvage chemotherapy. Apoptosis is a form of programmed cell death that occurs in multicellular organisms. It is well established that dysregulation of apoptosis plays an important role in pathogenesis of malignant diseases and may be associated with tumor progression and resistance to cytotoxic treatment. This study aimed to evaluate expression of apoptosis inducing factor (AIF) in GCTs. We observed lower AIF expression in GCTs compared to normal testicular tissue. We also showed prognostic significance of AIF in GCTs. AIF downregulation might represent one of the mechanisms of inhibition of apoptosis with subsequent facilitation of cell survival and metastatic dissemination of GCTs and perhaps could serve as a potential therapeutic target.

**Abstract:**

Apoptosis is a strictly regulated process essential for preservation of tissue homeostasis. This study aimed to evaluate expression of apoptosis inducing factor (AIF) in testicular germ cell tumors (GCTs) and to correlate expression patterns with clinicopathological variables. Formalin-fixed and paraffin-embedded specimens of non-neoplastic testicular tissue and GCTs obtained from 216 patients were included in the study. AIF expression was detected by immunohistochemistry, scored by the multiplicative quickscore method (QS). Normal testicular tissue exhibits higher cytoplasmic granular expression of AIF compared to GCTs (mean QS = 12.77 vs. 4.80, *p* < 0.0001). Among invasive GCTs, mean QS was the highest in embryonal carcinoma, yolk sac tumor and seminoma, lower in teratoma and the lowest in choriocarcinoma. No nuclear translocation of AIF was observed. Nonpulmonary visceral metastases were associated with lower AIF expression. Metastatic GCTs patients with high AIF expression had better overall survival compared to patients with low AIF expression (HR = 0.26, 95% CI 0.11–0.62, *p* = 0.048). We observed significantly lower AIF expression in GCTs compared to normal testicular tissue, which is an uncommon finding in malignant tumors. AIF downregulation might represent one of the mechanisms of inhibition of apoptosis and promotion of cell survival in GCTs.

## 1. Introduction

Testicular tumors represent a heterogenous group of neoplastic diseases accounting for approximately 1% of all malignant tumors in men. Although being relatively rare in general, it is the most commonly diagnosed cancer among men aged 15–40 years [1,2]. Histologically, germ cell tumors (GCTs) are the most common type [1]. In testes, postpubertal types of invasive GCTs (with the exception of spermatocytic tumor) are associated with a preinvasive lesion termed germ cell neoplasia in situ (GCNIS) [3]. GCTs may also primarily arise outside the gonads, most often in retroperitoneum or mediastinum, rarely elsewhere in the midline of the body or in its close proximity. It is presumed that this precise location is connected to migration of primordial germ cells into the genital ridge during embryogenesis [4,5]. Prognosis of GCTs is influenced by multiple factors, including histologic diagnosis of the tumor, its primary location, location of metastases, and clinical stage of the disease. Through identification of these parameters, patients with metastatic GCTs are categorized into ‘good’, ‘intermediate’ and ‘poor’ prognosis. This system proposed by International Germ Cell Cancer Collaborative Group (IGCCCG) in 1997 is beneficial for risk-based decisions in therapy [6,7]. Thanks to multimodal treatment, testicular cancer is considered the most curable solid neoplasm and patients with these tumors have the highest 5-year survival rates in general [7,8]. However, in small proportion of patients the disease progresses or relapses despite administration of salvage chemotherapy [9,10,11,12]. 

It is well established that dysregulation of apoptosis plays an important role in pathogenesis of malignant diseases and may be associated with tumor progression and resistance to cytotoxic treatment. Many neoplasms show abnormalities in expression of pro-apoptotic and antiapoptotic factors and a relationship between levels of these markers in tumor tissue and prognosis has been observed in many malignant diseases [13,14,15]. In testicular tumors, cisplatin-based chemotherapy is highly effective. Mechanisms of its action include, among other processes, activation of apoptotic pathways. Therefore, this type of cell death seems to be essential for cisplatin sensitivity [16,17].

Apoptosis-inducing factor (AIF) is a bifunctional flavoprotein exhibiting different actions according to its compartmentalization in the cell. It is capable of activating apoptotic process in a caspase-independent manner. Additionally, AIF has an important role in the maintenance of mitochondrial morphology and energy metabolism. The vital function of AIF requires its location in the intermembrane space of mitochondria. Upon apoptotic insult, AIF is translocated to the cytoplasm and subsequently, into the nucleus, where it exerts its lethal function through DNA fragmentation and chromatin condensation [18,19]. Preference of proapoptotic/antiapoptotic function of AIF seems to be tissue-specific. For instance, gene silencing of AIF which suppressed cisplatin-induced AIF expression in renal tubular cells leads to prolonged cell survival [20], while AIF gene knockout in colon cancer cell lines leads to increased sensitivity of tumor cells to peroxide- or drug-induced apoptosis [21]. Furthermore, the cytoplasmic form of AIF may interact with various cytoplasmic proteins which may also influence the pro- or antiapoptotic function of this molecule, since these reactions may either promote, suspend, or completely inhibit cell death [19]. Many studies have documented higher expression of AIF in malignant tumors compared to corresponding non-neoplastic tissues. Lewis et al. observed AIF overexpression in carcinoma of the prostate and significant reduction of growth and survival of tumor cells when AIF expression was suppressed. Since reduced cell survival correlated with decreased expression of mitochondrial complex I protein subunits, the authors stressed the importance of prosurvival function of AIF required for aggressive growth of the neoplasm [22]. AIF overexpression was also found in uveal melanoma [23], carcinoma of the stomach [24], colon [25], skin [26] and pancreas, chronic lymphocytic leukemia (CLL), and diffuse large B-cell lymphoma (DLBCL) [27,28,29]. On the other hand, downregulation of AIF is a rare event in malignant tumors. The research of Xu et al. revealed that expression of this molecule was significantly higher in normal noncancerous renal tissue in comparison with renal cell carcinoma. AIF expression was also lower in renal adenomas compared to normal kidney tissue. These results suggested tissue-specific function of AIF and indicated that AIF downregulation might play an important role in early stages of kidney tumor development [30]. Association between AIF expression and disease prognosis has been observed in various malignant tumors [23,28,29,31,32]. However, currently, prognostic value of AIF in testicular GCTs (TGCTs) remains unknown. 

## 2. Materials and Methods

### 2.1. Patients

This study included 216 patients with GCTs treated from January 1999 to December 2013 at National Cancer Institute and St Elizabeth Cancer Institute in Bratislava, Slovakia, tertiary comprehensive cancer centers. Paraffin-embedded tumor tissue samples and sufficient clinical data were available in all cases. Patients with concurrent malignancy other than nonmelanoma skin cancer in the previous 5 years were excluded. The data involving patient age, tumor histology, clinical stage, location and number of metastatic sites, delivery of systemic therapy, IGCCCG risk group and date of disease progression/death was registered and compared with AIF expression. All patients were treated with state-of-the-art treatment. The Institutional Review Board approved this retrospective study and a waiver of consent from patients was granted.

### 2.2. Diagnosis and Tissue Samples

Primary testicular GCT had 208 patients while the remainder was represented by biopsies of retroperitoneal and mediastinal masses in six and two cases, respectively. Invasive GCTs, GCNIS and non-neoplastic testicular tissue were evaluated in all cases, when available. GCTs were classified according to World Health Organization criteria [3]. Normal testicular tissue from noncancer patients was not available for analysis, therefore, we used non-neoplastic testicular tissue represented by seminiferous tubules with preserved spermatogenesis adjacent to testicular tumor which was identified in 64 cases. In total, 62 samples of GCNIS were also evaluated. The diagnosis of GCNIS was assessed through morphological criteria and OCT3/4 positivity. Tissue microarray was constructed as described previously [33].

### 2.3. Cell Lines

Human GCT cell lines NTERA-2 (embryonal carcinoma, ATCC^®^ CRL-1973TM), JEG-3 (choriocarcinoma, ATCC^®^ HTB-36™), NOY-1 (yolk sac tumor, catalog number: ENG101, FA: Kerafast), TCam-2 (seminoma, kindly provided by Dr. Kitazawa, Ehime University Hospital, Shitsukawa, Japan), and their cisplatin-resistant variants were used for the study. The cisplatin-resistant subclones (designed as CisR) were derived by propagating the cells in increasing concentrations of cisplatin (Hospira UK Ltd., Warwickshire, UK) for six months. NOY-1 and TCam-2 cells were maintained in RPMI (GIBCO^®^ Invitrogen, Carlsbad, CA, USA) and NTERA-2 and JEG-3 in DMEM (PAA Laboratories GmbH, Pasching, Austria) containing 10% FBS (GIBCO^®^ Invitrogen, Carlsbad, CA, USA), 10000 IU/mL penicillin (Biotica, Part. Lupca, Slovakia), 5 μg/mL streptomycin (Sigma-Aldrich, Saint-Louis, MO, USA), 2.5 μg/mL amphotericin (Sigma-Aldrich), and 2 mM glutamine (PAA Laboratories GmbH, Pasching, Austria). Cells were cultivated at 37 °C in humidified atmosphere and 5% CO_2_.

### 2.4. Western Blot

Cell pellets were resuspended in RIPA buffer (Cell Signaling Technology^®^, Danvers, MA, USA) containing Roche complete™ Protease Inhibitor Cocktail (Sigma-Aldrich) and the lysates were centrifuged for 10 min at 14,000× *g* at 4 °C. Concentration of protein in supernatants was determined using Modified Lowry Protein Assay Kit (Thermo Fisher Scientific Inc. Waltham, MA, USA). Electrophoresis on gradient SDS polyacrylamide gels was used for the separation of protein extract from each sample and proteins were then transferred to Hybond PVDF blotting membrane (GE Healthcare, Life Sciences, Chicago, IL, USA) using semidry blotting (Owl Inc., London, UK). One membrane was blocked in 5% nonfat dry milk in TBS-T for 1 h at room temperature and then incubated with primary mouse monoclonal AIF antibody overnight at 4 °C (sc-13116, Santa Cruz, Dallas, Texas, USA; dilution 1:250; 57 kDa). The second membrane was blocked in 5% nonfat dry milk in TBS-T overnight at 4 °C and then incubated with β-actin primary antibody (ab6276, Abcam, Cambridge, UK; dilution 1:5000; 42 kDa) for 1 h at room temperature. Horseradish peroxidase-linked secondary goat anti-mouse antibody (ab6789, Abcam, Cambridge, UK) and chemiluminescence detection system (Luminata™ Crescendo Western HRP Substrate, Millipore, Burlington, MA, USA) were used for the visualization. Each membrane was digitally captured with C-DiGit imaging system (LI-COR, Lincoln, NE, USA) and ratio AIF/β-actin was calculated using GelAnalyzer software (GelAnalyzer2010a (Available online: www.gelanalyzer.com (accessed on 20 January 202)) by Istvan Lazar Jr., PhD and Istvan Lazar Sr., PhD, CSc).

### 2.5. Gene Expression Analysis

Cultured cells were collected by trypsinization and total RNA was isolated by NucleoSpin^®^ RNA II (Macherey-Nagel, Düren, Germany) and treated with RNase-free DNase (Qiagen, Hilden, Germany). Total RNA was subjected to control PCR to confirm the absence of genomic DNA contamination. RNA was reverse transcribed with RevertAid™ H minus First Strand cDNA Synthesis Kit (Thermo Fisher Scientific Inc). Sensitive GCT cell lines as well as their resistant variants were subjected to quantitative RT PCR for AIFM1 gene using the TaqMan Gene Expression Assay (Applied Biosystems, Foster City, CA, USA). In brief, the total PCR reaction mixture (20.0 μL) contained 1.0 μL cDNA (75 ng), 10.0 μL TaqMan™ Fast Advanced Master Mix (Applied Biosystems), 8.0 μL nuclease-free water and 1.0 μL AIFM1 Taqman Gene Expression Assay (Hs00377585_m1, Applied Biosystems). Quantitative RT-PCR was carried out using the AriaMx Real-time PCR System (Agilent, Santa Clara, CA, USA). The thermal cycling program was 50 °C for 2 min for optimal UNG (Uracil N-glycosylase) activity, initial denaturation at 95 °C for 2 min followed by a 40 cycles two-step amplification consisting of denaturation at 95 °C for 3 s and annealing-cum-extension at 60 °C for 30 s. The obtained data were analyzed by Agilent Aria software version 1.5. Relative gene expression change was examined using the 2−ΔΔCt method. Expression levels were normalized to HPRT1 (Hs03929098_m1, Applied Biosystems) gene expression, which was used as the endogenous reference. All samples were analyzed in triplicate and data are expressed as means ± SEM. The significance of fold changes in gene expression between groups was analyzed using Student’s *t*-test applied to the ΔCt values.

### 2.6. Xenograft Model

Six- to eight-week-old NSG mice (The Jackson Laboratory, Bar Harbor, ME, USA) were used in accordance with institutional guidelines under approved protocols. Project was approved by the Institutional Ethic Committee and by the national competence authority (State Veterinary and Food Administration of the Slovak Republic), registration No. Ro 1030/18-221 in compliance with the Directive 2010/63/EU and the Regulation 377/2012 on the protection of animals used for scientific purposes. It was performed in the approved animal facility (license No. SK UCH 02017). 

Suspension of 2x106 GCT cells, both parental and resistant, in 100 µL of extracellular matrix (ECM) mixture 1:1 (50 µL serum free DMEM medium, 50 µL ECM) was injected s.c. into the flank of NSG mouse. Xenografts were measured by caliper and animals were sacrificed at the point when the tumors exceeded 1 cm in diameter. From tumor tissue formalin-fixed and paraffin-embedded specimens were prepared and further evaluated by immunohistochemistry.

### 2.7. Immunohistochemistry

Slides were deparaffinized, rehydrated, and immersed in phosphate buffered saline buffer (10 mM PO43-, 0.,9% NaCl, pH 7.,2). Tissue epitopes were demasked through revitalization in TRIS-EDTA retrieval solution (10 mM TRIS, 1 mM EDTA, pH 9.0) at 98 °C for 20 min in Dako PT Link (Dako, Glostrup, Denmark). The slides were subsequently incubated for 90 min at room temperature with primary mouse monoclonal antibody against AIF (AIF (E-1): sc-13116, Santa Cruz Biotechnology, Santa Cruz, CA, USA) diluted 1:500 in Dako REAL antibody diluent (Dako) and immunostained using anti-mouse/anti-rabbit secondary antibody (EnVision FLEX / HRP, Dako) for 30 min at room temperature. The reaction was visualized by diaminobenzidine substrate-chromogen solution (DAB, Dako) which was applied for 5 min. Ultimately, the slides were counterstained with hematoxylin. Non-neoplastic testicular tissue was used as a positive control and the same tissue without incubation in primary antibody represented the negative control.

### 2.8. Immunohistochemical Stain Scoring

Tumor cores were independently evaluated by three observers (KL, PB and PJ) who were blinded to clinicopathological data. Xenograft model tumors were evaluated by two observers (ZC, SH). In the case of disagreement, the result was reached by consensus. AIF expression was scored by the multiplicative quickscore (QS) method which accounts both for percentage of positive cells and staining intensity as described previously [33]. Based on QS value, AIF expression was categorized as either low (QS = 0–9) or high (QS = 10–18).

### 2.9. Statistics

Shapiro–Wilk normality test was used to determine whether AIF QS values were normally distributed. Since the distribution of the variables was significantly different from normal distribution, we used nonparametric tests for further analysis. Mann–Whitney U test was used for analysis of differences in distribution of AIF expression between two groups of patients. If more than two groups of patients or samples were compared, Kruskal–Wallis test was carried out. In the case of categorization of AIF expression to ‘low’ or ‘high’ according to criteria described above, Fisher’s exact test or χ2 test were applied.

The median follow-up period was calculated as a median observation time among all patients and among those still alive at the time of their last follow-up. Progression-free survival (PFS) was calculated from the date of orchiectomy or the date of tumor biopsy to the date of progression or death or the date of the last adequate follow-up. Overall survival (OS) was calculated from the date of orchiectomy or the date of tumor biopsy to the date of death or the last follow-up. PFS and OS were estimated using the Kaplan–Meier product limit method and compared by the log-rank test. Multivariate Cox proportional hazards model for PFS and OS was used to assess differences in outcome on the basis of AIF expression in primary tumor and prognosis according to IGCCCG (International Germ Cell Collaborative Group) [6]. For all statistical analyses, a *p*-value of < 0.05 was considered significant. Statistical analyses were performed using NCSS 2019 software (NCSS 2019 Statistical Software. 2019, NCSS, LLC: Kaysville, Utah, USA).

## 3. Results

### 3.1. AIF Expression Analysis in Germ Cell Cancer Cell Lines

**Figure 1:**
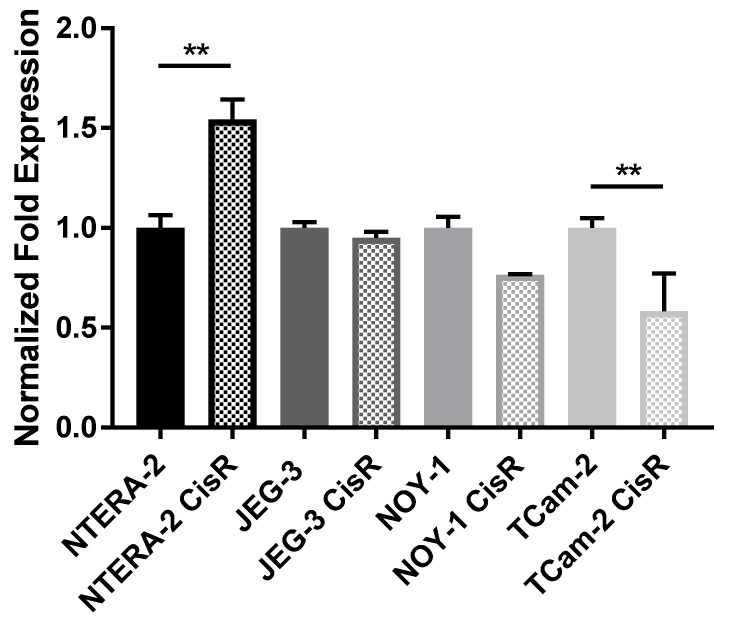
Relative apoptosis-Inducing Factor (AIF) expression in different GCTs cell lines on mRNA level by qRT-PCR. ** *p* < 0.01

**Figure 2:**
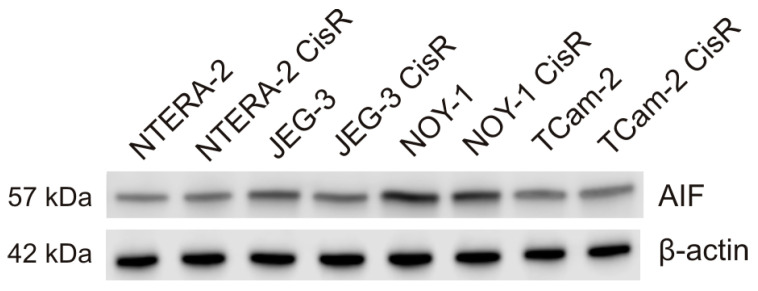

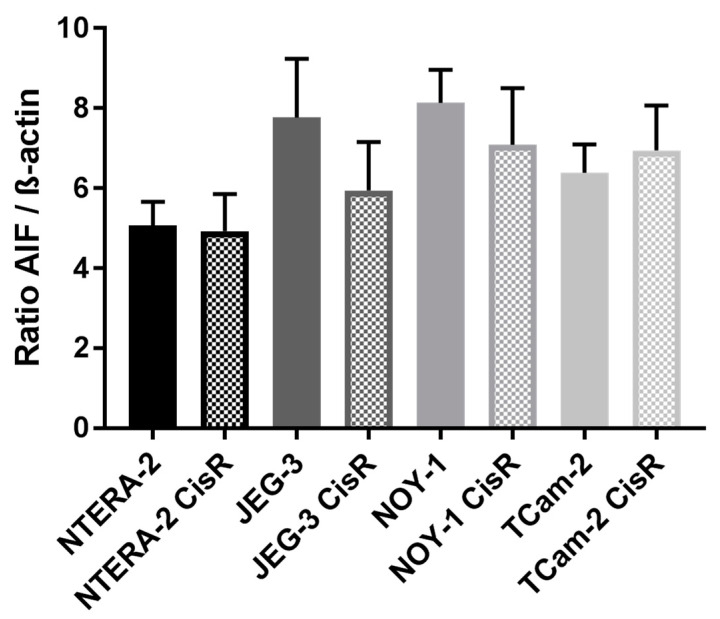
AIF expression in different GCTs cell lines on protein level by Western blot.

**Figure 3:**
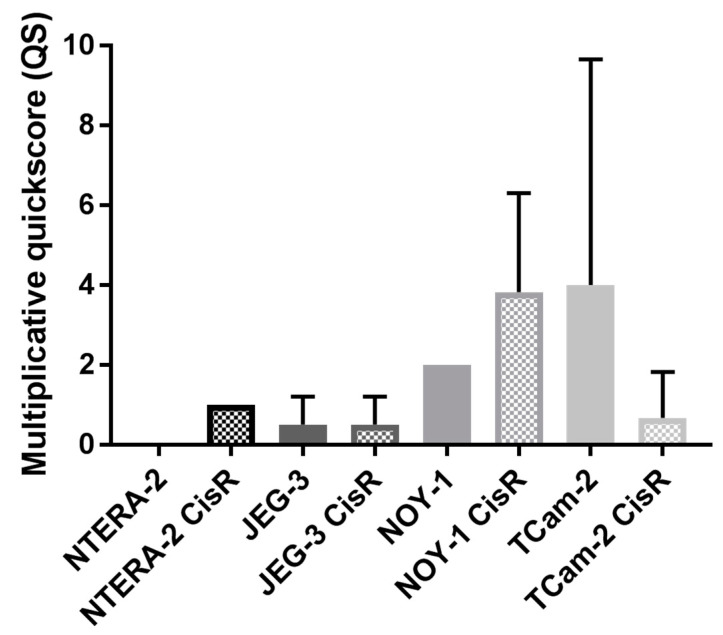
AIF expression in different GCTs cell lines in xenograft model.

Using quantitative RT-PCR we detected significant differences in AIF expression between cisplatin sensitive and resistant GCT cell lines NTERA-2 and TCam-2. While cisplatin resistant cells NTERA-2 showed significant higher AIF expression when compared to sensitive cells (*p* = 0.00118), in seminoma cell line TCam-2 significant higher AIF expression on mRNA level was detected in sensitive cells (*p* = 0.00369) (Figure 1). However, there were no differences in AIF expression on protein level between cisplatin sensitive or resistant GCTs cell lines, nor between cell lines derived from different histological subtype of GCTs (Figure 2 and Supplementary Figure S1). Numerically, on protein level the highest expression was observed in choriocarcinoma JEG-3 cells. In germ cell cancer xenograft model using these cell lines, AIF expression was consistent with qRT-PCR results, with higher expression of AIF in cisplatin resistant cells NTERA-2 and TCam-2 sensitive cell lines (Figure 3). Moreover, cisplatin resistant NOY-1 exhibited increased AIF expression on mRNA level as well.

### 3.2. AIF Expression in GCTs

**Figure 4:**
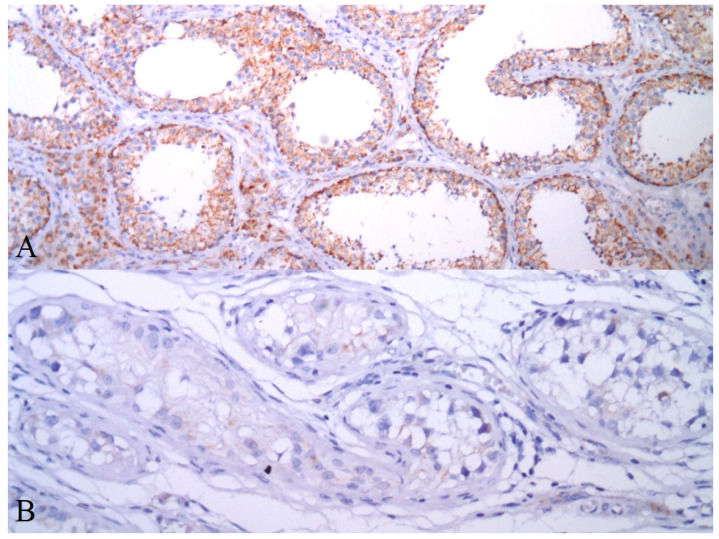
AIF expression (immunoperoxidase technique) in spermatogenic cells of noncancerous testicular tissue (**A**, 100×) and in GCNIS (**B**, 200×).

**Figure 5:**
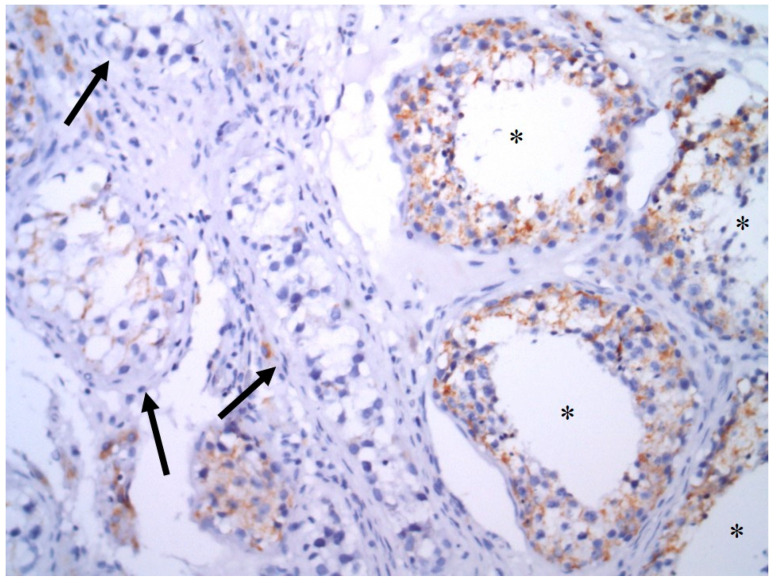
AIF expression in in spermatogenic cells of noncancerous testicular tissue (*) and in GCNIS (arrows) (immunoperoxidase technique, 200×).

**Figure 6:**
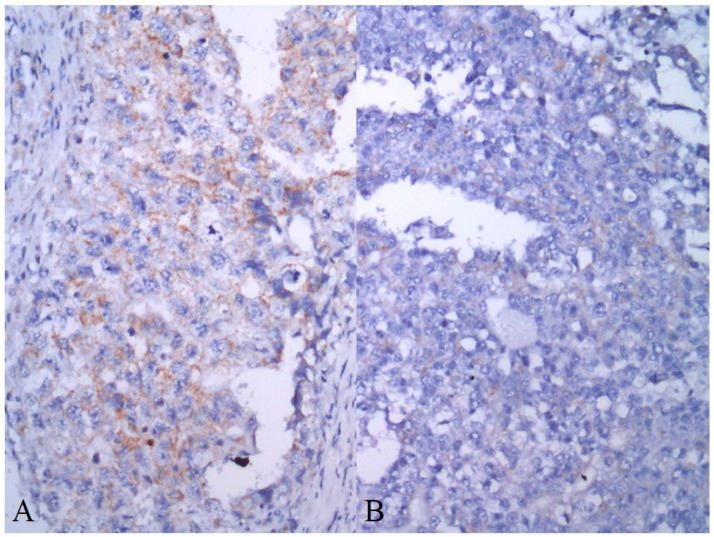
High (**A**) and low (**B**) AIF expression in two different samples of embryonal carcinoma (immunoperoxidase technique, 200×).

**Figure 7:**
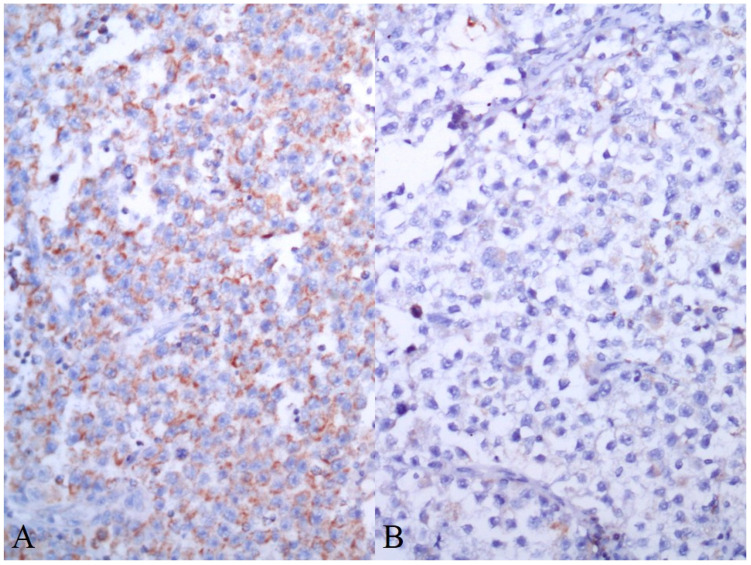
High (**A**) and low (**B**) AIF expression in two different samples of seminoma (immunoperoxidase technique, 200×)**.**

Patient characteristics are summarized in Table 1. AIF expression in GCTs is summarized in Table 2. Seventy-four (34.3%) of the evaluated tumors were pure invasive GCTs, while 142 (65.7%) tumors represent mixed GCTs. Cytoplasmic granular positivity of AIF exhibited 151 (69.9%) of the evaluated samples. AIF expression was significantly higher in spermatogenic cells of noncancerous testicular tissue compared to germ cell tumors (mean QS ± standard error of the mean (SEM) = 12.77 ± 0.65 versus 4.80 ± 0.31, *p* < 0.0001) and GCNIS (mean QS ± SEM = 5.66 ± 0.67, *p* < 0.0001) (Figure 4 and Figure 5). As much as 78.1% of normal testicular tissue samples, yet only 19.4% of GCTs, exhibited high QS (QS ≥ 10). Among GCTs, the highest AIF expression was observed in GCNIS, with decreasing values of mean QS in embryonal carcinoma (EC) (Figure 6), yolk sac tumor (YST), seminoma (SE) (Figure 7), epithelial teratoma, mesenchymal teratoma, and choriocarcinoma (CHC). Detectable nuclear translocation of AIF was not observed. 

### 3.3. Association between Various Patient/Tumor Characteristics and AIF Expression in GCTs

Analysis of association between various patient/tumor characteristics and AIF expression in GCTs is shown in Table 3. AIF expression was significantly higher in nonseminomatous tumors compared to seminomas (mean QS ± SEM = 5.07 ± 0.35 vs. 3.63 ± 0.72, *p* = 0.04). AIF expression was numerically lower in primary extragonadal GCT in comparison with primary testicular GCTs, though the difference was not statistically significant (mean QS ± SEM = 3.88 ± 1.63 vs. 4.83 ± 0.32, *p* = 0.40). In the group of patients with poor prognosis according to IGCCCG and more advanced disease (including patients with three or more metastatic sites, mediastinal lymph node metastases, liver metastases, or presence of other nonpulmonary visceral metastases) we observed lower AIF expression in primary tumor compared to patients with good/intermediate prognosis and less advanced disease (Table 3). However, the differences were not statistically significant except of patients with nonpulmonary visceral metastases (NPVM) that had low AIF expression compared to patients without NPVM (QS = 2.93 vs. 4.96, *p* = 0.05).

### 3.4. Prognostic Value of AIF in Testicular Germ Cell Tumors

**Figure 8:**
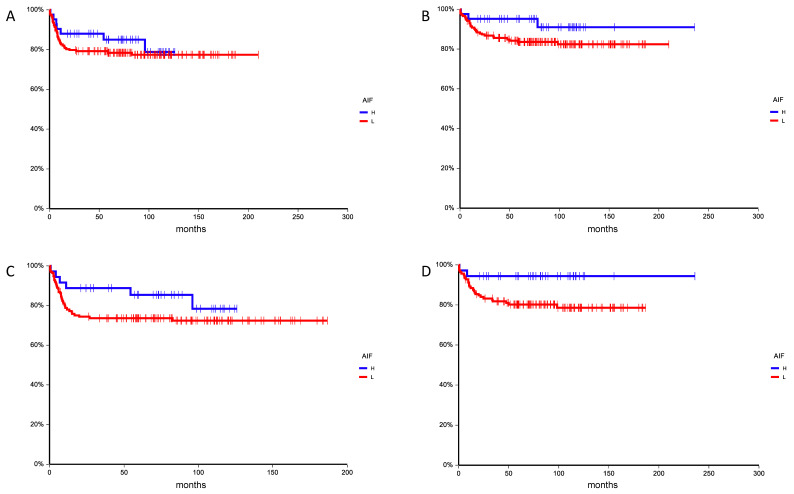
(**A**) Kaplan–Meier estimates of probabilities of progression-free survival according to AIF expression in testicular germ cell tumor patients (*n* = 216), HR = 0.74, 95% CI 0.35–1.53, *p* = 0.46; H—high AIF (QS ≥ 10); L—low AIF (QS < 10). (**B**) Kaplan–Meier estimates of probabilities of overall survival according to AIF expression in testicular germ cell tumor patients (*n* = 216), HR = 0.42, 95% CI 0.18–1.00, *p* = 0.14; H—high AIF (QS ≥ 10); L—low AIF (QS < 10). (**C**) Kaplan–Meier estimates of probabilities of progression-free survival according to AIF expression in metastatic testicular germ cell tumor patients (stage IS to III.C) (*n* = 173), HR = 0.57, 95% CI 0.28–1.18, *p* = 0.20; H—high AIF (QS ≥ 10); L—low AIF (QS < 10). (**D**) Kaplan–Meier estimates of probabilities of overall survival according to AIF expression in metastatic testicular germ cell tumor patients (stage IS to III.C) (*n* = 173), HR = 0.26, 95% CI 0.11–0.62, *p* = 0.048; H—high AIF (QS ≥ 10); L—low AIF (QS < 10).

The median follow-up time was 81.1 months (0.3–235.8 months) for all patients and 93.8 months (21.0–235.8 months) for patients still alive. During follow-up, 46 (21.3%) patients experienced disease progression and 32 (14.8%) patients died. All observed deaths were due to testicular cancer. The estimated 5-year PFS and OS was 79.7% (95% CI 74.3–85.2%) and 85.8% (95% CI 81.0%–90.5%), respectively. Patients with high AIF (QS ≥ 10) expression in primary tumor had better PFS when compared to patients with low AIF (QS < 10) expression (hazard ratio (HR) = 0.74, 95% CI 0.35–1.53, *p* = 0.46) (Figure 8A). Similarly, patients with high AIF had better OS than patients with low AIF (HR = 0.42, 95% CI 0.18–1.00, *p* = 0.14) (Figure 8B). 

In patients with metastatic disease (stage from IS to III.C), patients with high AIF (QS ≥ 10) expression in primary tumor had better PFS when compared to patients with low AIF (QS < 10) expression (hazard ratio (HR) = 0.57, 95% CI 0.28–1.18, *p* = 0.20) (Figure 8C). Similarly, patients with high AIF had better OS than patients with low AIF (HR = 0.26, 95% CI 0.11–0.62, *p* = 0.048) (Figure 8D). However, in multivariate analysis, AIF expression in primary tumor was not associated with OS independently of IGCCCG risk group (HR = 0.37, 95% CI 0.09–1.60, *p* = 0.19).

## 4. Discussion

In this study, we observed significantly higher AIF expression in non-neoplastic testicular tissue compared to both invasive GCTs and GCNIS. Totally, 78.1% of the samples with normal seminiferous tubules but only 19.4% of GCTs showed AIF overexpression. Since malignant tumors of various primary locations and histologic subtypes exhibit mostly elevated AIF expression in comparison with corresponding normal tissues, the finding of low AIF expression in GCTs compared to normal testicular tissue is surprising. This observation might be associated with dual function of this protein. On one hand, AIF is important for preservation of morphologic integrity and functioning of the cell, but on the other hand, under certain circumstances, it represents a lethal signal and after translocation to the cytoplasm, it is relocated to the cell nucleus and causes degradation of DNA [19]. 

Lee et al. and Cobanoglu et al. attributed high AIF expression in colon cancer cells to the prosurvival function of mitochondrial form of this protein associated with its oxidoreductase activity which might participate in tumorigenesis, metastatic spread, and resistance to cytotoxic treatment [24,34]. Similar interpretation was used in studies of AIF expression in other neoplasms; AIF was perceived as an element significant for survival of cancer cells and progression of oncologic diseases [23,24,26,27,28]. Moreover, inactivation of AIF in pancreatic and colon cancer cell lines leads to their enhanced apoptosis sensitivity and growth damage [21,27]. In a study conducted by Li et al., AIF expression was lower in small lymphocytic lymphoma/CLL compared to DLBCL. In the majority of DLBCL samples, strong AIF positivity was detected in the cytoplasm of the tumor cells. DLBCL is an aggressive type of lymphoma with rapid progression. Due to cytoplasmic location of positive immunohistochemical reaction, the authors speculated about the presence of an unknown factor that might inhibit translocation of apoptogenic AIF to the nucleus with resulting inhibition of apoptosis and aggressive biological behavior of the neoplasm. They added that nuclear expression of AIF, which was not detected in any of the samples, may be a temporary phenomenon [35]. On the contrary, decline in AIF expression is not common in malignant tumors. Xu et al. reported significant downregulation of this protein in certain types of benign and malignant tumors of the kidney, while cytoplasmic positivity of AIF in normal renal tubules was strong and diffuse. Furthermore, forced expression of AIF in renal cell carcinoma cell lines leads to massive apoptosis [30]. 

In our study, we observed decreased AIF expression in GCTs compared to normal seminiferous tubules. AIF positivity was exclusively located in the cell cytoplasm, which might result from detection of the mitochondrial form of this protein. This could explain far higher expression in normal testicular tissue where AIF might exert its prosurvival, antiapoptotic function. However, cells of GCTs are considered extremely prone to apoptosis induced by DNA damage [16]. Therefore, the unique sensitivity to apoptosis in GCTs might be explained by transformation of a large pool of mitochondrial AIF to proapoptotic variant of this protein upon apoptogenic insult. As previously conducted studies suggest, the role of AIF in tumorigenesis seems to be tissue-specific and absence of nuclear AIF positivity does not necessarily rule out detection of its proapoptotic form [20,21,30,35]. Thus, downregulation of AIF in GCTs may be associated with inhibition of apoptosis and promotion of cell proliferation and metastatic spread of the primary tumor. 

Interestingly, among all subtypes of GCTs, AIF expression was the lowest in choriocarcinomas (mean QS = 0.17) and teratomas (epithelial component: mean QS = 1.62, mesenchymal component: mean QS = 0.47). Choriocarcinoma is known for its rapid progression, widespread and early formation of metastases, and adverse prognosis despite intensified anticancer therapy. Teratoma is considered chemoresistant and therefore commonly found in specimens of residual tumor masses surgically removed after completion of chemotherapy [16]. It is well established that AIF participates in p53-induced apoptosis and cisplatin reaches its effect through this particular protein [36,37]. Consequently, low AIF expression in these tumor subtypes might be connected to their aggressive behavior and chemoresistance. Surprisingly, in experimental in vitro model, we did not observe differences in AIF expression on protein level between sensitive and resistant cell lines. NTERA-2 CisR exhibit higher AIF expression on mRNA level, while in TCam-2 the expression was opposite. This observation was consistent between qRT-PCR results and xenograft model. Analyzed patients’ samples were all chemotherapy naïve, and there were no primary cisplatin resistant tumors in the study population compared to GCTs cell lines. 

Previously reported data on prognostic significance of AIF is variable and inconsistent, presumably due to complex function of the molecule. In some studies, AIF overexpression was associated with better prognosis and longer survival of the patients, such as in DLBCL [29] and nonsmall cell lung cancer [32]. On the contrary, Krasnik et al. observed association of high AIF expression with certain unfavorable prognostic features and shorter survival of the patients with uveal melanoma [23]. In a study conducted by Jeong et al., cancer cells of colorectal carcinoma samples exhibited higher intensities of AIF immunostaining than normal mucosal epithelial cells, but immunoreactivities were present in the cancer irrespective of their location or depth of invasion [25]. Similarly, no significant association between AIF expression and depth of invasion nor any considerable differences in AIF expression between histologic subtypes were observed in gastric carcinoma [24].

In our study, we observed numerically lower AIF expression in extragonadal GCTs compared to GCTs. Higher levels of AIF were present in poor prognosis patients and in patients with more advanced disease, including patients with metastases in mediastinal lymph nodes, liver and other nonpulmonary visceral metastases and stage III patients. Low AIF expression in primary tumor was therefore associated with some adverse prognostic factors; however, the differences were not statistically significant. We speculate that association of low AIF expression with unfavorable prognostic features might be connected to inhibition of apoptosis in cells of GCTs caused by decrease of levels of AIF with consecutive prolongation of cell survival, facilitation of metastatic dissemination, and perhaps, also resistance to chemotherapy. In the group of patients with metastatic disease, patients with high AIF expression in primary tumor had better OS when compared to patients with low expression of this protein. However, presumably due to correlation between AIF expression and some prognostic features used in classification of the patients to IGCCCG risk groups, AIF expression in primary tumor was not associated with OS completely independently.

## 5. Conclusions

In conclusion, our study documented lower AIF expression in GCTs compared to normal testicular tissue, which, in general, is a rare event in malignant tumors. We suppose that this might be related to a tissue-specific effect of AIF and its decrease may be involved in development of GCTs. AIF downregulation might represent one of the mechanisms of inhibition of apoptosis with subsequent facilitation of cell survival and metastatic dissemination of GCTs and perhaps could serve as a potential therapeutic target. We also showed prognostic significance of AIF in GCTs. However, further research is needed to elucidate the role of AIF in pathogenesis of testicular neoplasms and to determine whether AIF is also a predictive factor with possible impacts on targeted therapy of GCTs. Apoptosis-targeting agents are already in clinical development for cancer research and further research should focus on evaluating efficacy of these agents alone or in combination of currently used chemotherapy aimed to reverse treatment resistance.

## Figures and Tables

**Table 1:** Patient characteristics.

Variable	N	%
**All**	216	100.0
**Histology**		
Seminoma	41	19.0
Nonseminoma	175	81.0
**Primary tumor**		
Gonadal	208	96.3
Retroperitoneal	6	2.8
Mediastinal	2	0.9
**IGCCCG risk group**		
Good prognosis	163	75.5
Intermediate prognosis	24	11.1
Poor prognosis	29	13.4
**Sites of metastases**		
Retroperitoneum	154	71.3
Mediastinum	22	10.2
Lungs	50	23.1
Liver	12	5.6
Brain	1	0.5
Visceral nonpulmonary metastases	15	6.9
**Number of metastatic sites**		
0	55	25.5
1	94	43.5
2	30	13.9
≥3	37	17.1
	Median	Range
**Patient age**	30	17–67
**Pretreatment level of tumor markers**		
AFP (mIU/mL)	7	0–60,570
HCG (IU/mL)	4	0–929,000
LDH (mkat/l)	6	2–89

AFP—alpha-fetoprotein; HCG—human chorionic gonadotropin; IGCCCG—International Germ Cell Collaborative Group; LDH—lactate dehydrogenase.

**Table 2:** AIF expression in primary germ cell tumors.

Histologic Subtype	Number of Samples (N)	Mean QS	SEM	Median	*p*-Value ^a^	Low QS ^b^		High QS ^c^		*p*-Value ^a^
						N	%	N	%	
Normal testis	64	12.77	0.65	13.5	NA	14	21.9	50	78.1	NA
Germ cell tumors	216	4.80	0.31	4.0	<0.0001	174	80.6	42	19.4	<0.0001
Seminoma	70	3.94	0.55	2.1	<0.0001	57	81.4	13	18.6	<0.0001
Embryonal carcinoma	128	5.17	0.40	4.0	<0.0001	102	79.7	26	20.3	<0.0001
Yolk sac tumor	26	4.73	0.83	4.0	<0.0001	23	88.5	3	11.5	<0.0001
Choriocarcinoma	12	0.17	0.17	0.0	<0.0001	12	100.0	0	0.0	<0.0001
Teratoma (epT)	21	1.62	0.65	0.0	<0.0001	21	100.0	0	0.0	<0.0001
Teratoma (mesT)	32	0.47	0.38	0.1	<0.0001	31	96.9	1	3.1	<0.0001
GCNIS	62	5.66	0.67	3.0	<0.0001	50	80.6	12	19.4	<0.0001

^a^ compared to normal testicular tissue, ^b^ QS = 0–9, ^c^ QS = 10–18, NA—not applicable; GCNIS—germ cell neoplasia in situ.

**Table 3:** Association between AIF expression in primary tumor and patients/tumor characteristics.

	AIF Expression									
Variable	Number of Patients (N)	Mean QS	SEM	Median	*p* Value ^a^	Low QS ^b^		High QS ^c^		*p* Value ^a^
						N	%	N	%	
**All**	216	4,80	0,31	4.0	NA	174	80.6	42	19,4	NA
**Histology**										
Seminoma	41	3.63	0.72	2.0	0.04	34	82.9	7	17.1	0.83
Nonseminoma	175	5.07	0.35	4.0		140	80.0	35	20.0	
**Tumor primary**										
TGCT	208	4.83	0.32	4.0	0.40	167	80.3	41	19.7	1.00
EGCT	8	3.88	1.63	1.0		7	87.5	1	12.5	
**IGCCCG risk group**										
Good prognosis	163	5.01	0.36	4.0	0.07	129	79.1	34	20.9	0.12
Intermediate prognosis	24	5.50	0.94	4.0		18	75.0	6	25.0	
Poor prognosis	29	3.03	0.85	2.0		27	93.1	2	6.9	
**Number of metastatic sites**										
0	55	4.85	0.62	4.0	0.45	47	85.5	8	14.5	0.24
1–2	130	5.04	0.40	4.0		100	76.9	30	23.1	
≥3	31	3.80	0.84	3.0		27	90.0	4	13.3	
**Retroperitoneal LN metastasis**										
Present	154	4.86	0.37	4.0	0.94	120	77.9	34	22.1	0.13
Absent	62	4.63	0.59	4.0		54	87.1	8	12.9	
**Mediastinal LN metastases**										
Present	22	3.68	0.33	4.0	0.13	18	81.8	4	18.2	1.00
Absent	193	4.95	0.98	1.0		155	80.3	38	19.7	
**Lung metastases**										
Present	50	4.92	0.65	4.0	0.91	40	80.0	10	20.0	1.00
Absent	165	4.79	0.36	4.0		133	80.6	32	19.4	
**Liver metastases**										
Present	12	3.08	1.33	3.5	0.28	12	100.0	0	0.0	0.13
Absent	203	4.92	0.32	4.0		161	79.3	42	20.7	
**Non-pulmonary visceral metastases**										
Present	15	2.93	1.19	3.0	0.17	15	100.0	0	0.0	0.05
Absent	200	4.96	0.32	4.0		158	79.0	42	21.0	
**Stage**										
0	81	4.68	0.51	4.0	0.38	66	81.5	15	18.5	0.50
I	83	5.42	0.51	4.0		63	75.9	20	24.1	
II	30	4.53	0.84	3.0		25	83.3	5	16.7	
III	19	3.58	1.06	0.0		17	89.5	2	10.5	

^a^ compared to normal testicular tissue, ^b^ QS = 0–9, ^c^ QS = 10–18, NA—not applicable; GCNIS—germ cell neoplasia in situ; TGCT—testicular germ cell tumor; EGCT—extragonadal germ cell tumor; LN—lymph node.

## Data Availability

The data presented in this study are available on request from the corresponding author.

## Supplementary Materials

The following are available online at https://www.mdpi.com/2072-6694/13/4/776/s1, Figure S1: Western blot analysis of AIF and β-actin levels in different GCT cell lines.
