# Supplementary material for: Prognostic Value of Apoptosis-Inducing Factor (AIF) in Germ Cell Tumors
Source: Cancers (Basel). 2021 Feb 13;13(4):776. doi: 10.3390/cancers13040776 (PMC7917670; doi:10.3390/cancers13040776)
Supplement: Supplementary file 1 [file cancers-13-00776-s001.pdf]

Supplementary materials

Prognostic Value of Apoptosis-Inducing Factor (AIF) in Germ Cell Tumors

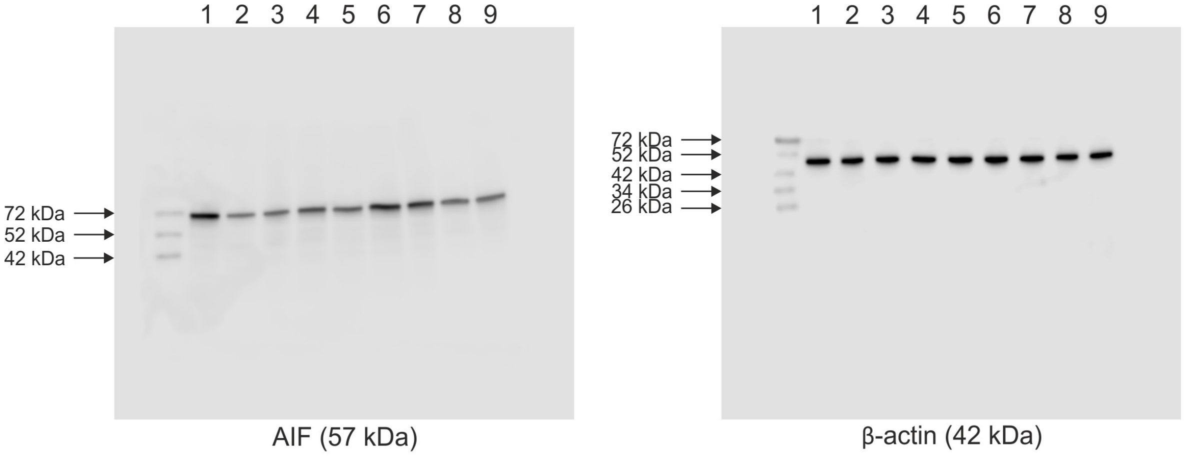

	Sample	AIF intensity	ß-actin intensity	AIF/ß-actin ratio
1	positive control	8675	10389	8,35
2	NTERA-2	4324	9848	4,39
3	NTERA-2 CisR	4842	10378	4,67
4	JEG-3	6613	10244	6,46
5	JEG-3 CisR	5951	10571	5,63
6	NOY-1	8600	10981	7,83
7	NOY-1 CisR	7672	10402	7,38
8	TCam-2	5242	9408	5,57
9	TCam-2 CisR	5825	9470	6,15

Supplementary Figure S1. Western blot analysis of AIF and β-actin levels in different GCT cell lines. AIF and β-actin intensities, and AIF/β-actin ratios for each cell line are listed in the table.
